# Cyclosporin A Prevents Ovarian Graft Rejection, and Permits Normal Germ Cell Maturation Within the First 5 Weeks Post-transplantation, in the Domestic Turkey (*Meleagris gallopavo*)

**DOI:** 10.3389/fvets.2022.855164

**Published:** 2022-04-15

**Authors:** George B. Hall, Janet Beeler-Marfisi, Julie A. Long, Benjamin J. Wood, Gregoy Y. Bedecarrats

**Affiliations:** ^1^Department of Animal Bioscience, Ontario Agriculture College, University of Guelph, Guelph, ON, Canada; ^2^Department of Pathobiology, Ontario Veterinary College, University of Guelph, Guelph, ON, Canada; ^3^Animal Biosciences and Biotechnology Laboratory, Beltsville Agricultural Research Center, Agricultural Research Service, United States Department of Agriculture (USDA), Beltsville, MD, United States; ^4^School of Veterinary Medicine, The University of Queensland, Gatton, QLD, Australia

**Keywords:** turkey, ovary, transplantation, immunosuppressant, lymphocyte

## Abstract

Biobanked ovaries collected from recently hatched poults can only be revived through transplantation, using a recipient bird. The main hurdle in transplantation is preventing graft rejection, which appears as lymphocytic infiltration upon histologic evaluation of the graft. In this study, the condition of the transplants [immunological compatibility (auto- vs. allotransplants), donor age, time in holding media, and temperature of holding media] and treatment of recipient poults with varying immunosuppressants [mycophenolate mofetil (MFM), cyclophosphamide (CY), and cyclosporin A (CsA)] were studied to determine which factors could reduce lymphocytic infiltration, during the first 35 days post-transplantation. Lymphocytic infiltration was determined *via* cytoplasmic CD3 (T cell) and nuclear PAX5 (B cell) expression. There was no significant difference in the percent of cytoplasmic CD3 or nuclear PAX5 immunostained area between the unoperated group and the autotransplants, by 6 days post-transplantation. However, the allotransplants had more (*P* < 0.05) positive cytoplasmic and nuclear immunostained areas compared to autotransplants, irrespective of donor age, time in holding media or temperature of the media. By 14 days post-transplantation, the CsA 25 and 50 mg/kg/day treatment groups had less (*P* < 0.05) CD3 and PAX5 positive areas in their allotransplants, compared to the unsuppressed group. At 35 days post-transplantation, the CsA 25 mg/kg/day allotransplant group also had less (*P* < 0.05) CD3 and PAX5 positive areas compared to the unsuppressed group. The CsA 25 mg/kg/day transplants also had a similar ovarian follicular size compared to the unoperated group, although they contained fewer (*P* < 0.05) follicles based on follicular density. Donor age, duration in holding media, temperature of media, and treatment of recipients with MFM or CY had no effect on reducing lymphocytic infiltration. However, immunological compatibility was associated with decreased lymphocytic infiltration, as autotransplants had little lymphocytic infiltration. Treatment of recipients with CsA at 25 mg/kg/day was also associated with reduced lymphocytic infiltration and allowed transplants to develop normally during the first 35 days post transplantation.

## Introduction

To preserve and protect the genetics and biodiversity of poultry breeds and lines, two main methods of biobanking have been proposed. The first corresponding to cryopreservation of primordial germ cells (PGCs) either on their own (1) or held within an immature gonad (2), and the second relying on the combined cryopreservation of post-hatch ovaries and testes, or sperm, from mature males (3–5). Both techniques preserve the full genome of the breed or line, which can be revived within one generation. However, manipulation of PGCs is technically challenging and costly, and collection is limited to a small window between 50 and 56 h of embryonic development. Alternately, collection of the ovary or testes from post-hatch chicks and sperm from mature males, is simpler, less expensive, and gonads can be collected from day old chicks to mature individuals (6, 7). This increases the age range of individuals that could be biobanked. Thus, the latter technique has a broader applicability, especially for rare and endangered poultry breeds, and their wild counterparts.

Although biobanking of sperm has been optimized for some avian species (8, 9), reliable cryopreservation and transplantation of ovarian tissue requires further optimization, especially in the domestic turkey. To date, ovarian transplant success rates in poultry breeds vary greatly between and within species and are technically challenging for routine use after biobanking (10–12). Thus, to ensure this technique can be used in the future to conserve poultry genetics, it is paramount to identify the factors that negatively influence transplant success rates in each species of interest, and to find remedies to counter those negative factors.

Our group has been focussing on possible reasons for ovarian transplant failure in the domestic turkey. In previous work, we elucidated the impact of age and ovarian developmental stage of the donor (13), along with the amount of recipient ovarian tissue which should remain to improve attachment rates (12). From these studies, we recommended that 7 days posthatch (dph) donor ovarian tissue is optimal as the ovary has few follicles, and that the entire recipient ovary should be removed, with the abdominal air sac membrane not used to cover the transplants. However, in these studies we also noted that by 6 days post-transplantation, the ovarian transplants were highly infiltrated by T and B cells, which indicated potential graft rejection. Therefore, to increase the success rate of ovarian transplantation, the current study focused on reducing or eliminating lymphocytic infiltration.

Factors that reduce lymphocytic infiltration and promote transplant survival can be categorized into two groups. These groups are pre-surgery transplant condition, and post-surgical treatment of the recipient. For pre-surgical condition of gonadal tissue, immunological compatibility (allo- vs. autotransplants), has had one of the strongest effects on increased survival rate of transplanted gonadal tissue (10, 14). Marked differences in transplant survival rates between day old ovaries (11) and mature ovaries was also noted (7), suggesting that donor age might be another pre-surgery condition that affects the survival rate. Finally, poultry gonadal tissue is routinely chilled pre-surgery (4, 11) and, although this has not been demonstrated to improve transplant survival rates, it is a common practice in mammalian whole organ transplantation, which has been proven effective in increasing transplant survival rates (15).

Immunosuppressants are the most common form of post-surgical treatment to improve transplant survival. They work to stop the host's immune response against the foreign tissue. In avian species, mycophenolate mofetil (MFM) was first used in ovarian transplantation between chicken breeds, but it did not improve graft success (14, 16). However, MFM did increase the number of successful transplants in quail (17). Dexamethasone was also used in poultry to suppress the immune system after ovarian transplantation; however, dexamethasone did not improve transplant success rates similar to MFM (11, 14). The immunosuppressants cyclophosphamide (**CY**) and cyclosporin A (**CsA**) have been extensively used in avian species to suppress lymphocytic numbers in virological and bacteriological studies (18–22). There has been one successful study which used CsA on chickens that received allotransplanted skin (18). However, these compounds have not been evaluated in poultry ovarian transplantation studies.

To elucidate the cause of ovarian graft rejection in turkeys, we investigated whether condition or pre-treatment of the transplants could reduce lymphocytic infiltration. These factors included immunological compatibility, age of the donor, time in holding media, and temperature of holding media. Effectiveness was measured by determining the area of cytoplasmic CD3- (T cell marker) and nuclear PAX5- (B cell marker) positivity within the transplants 6 days post-surgery. Next, three different immunosuppressants (MFM, CY, and CsA) were tested at different doses and administration routes during the first 14 days post-surgery. The area of CD3- and PAX5-positive immunostaining was again used as a marker of lymphocytic infiltration in transplants. Effectiveness of immunosuppression was also measured by lymphoid organ relative weights and concentration of peripheral white blood cells. The most effective immunosuppressive treatment was repeated and analyzed until 5 weeks post-surgery. Finally, to determine if the tissue was developing normally, ovarian follicular size and density were also assessed at 35 days post-transplantation.

## Materials and Methods

### Animals

All experimental and surgical procedures followed the approved Animal Utilization Protocol (#3921) granted by the Institutional Animal Care Committee who adhered to the principles described by the Canadian Council on Animal Care. All animals were housed in the University of Guelph's Animal Care Facility. Recipient and donor female poults were supplied by Hybrid Turkeys at 1 dph (Kitchener, Ontario Canada) from a converter parent stock line. Hence, all recipients and donors were from the same genetic population. Donor poults were given food and water *ad libitum* and were housed in groups. In trial 1, donor poults were euthanized at 2 or 9 dph *via* manual cervical dislocation, whereas, in trial 2 and 3 they were 7–9 dph. In all the trials the recipient poults were 2 dph. Prior to surgery, recipient poults were only given water and were kept in groups. Post-surgery, recipient poults were offered food and water *ad libitum* and were housed with donor poults of a similar age to ensure recipients quickly became familiar with their feed. Recipient poults were weighed daily to monitor weight gain and development until 8, 16 or 37 dph (i.e., 6-, 14- or 35-days post-transplantation, respectively) when they were euthanized by manual cervical dislocation.

### Experimental Design and Immunosuppressants

#### Study 1

In this study, four factors hypothesized to influence lymphocytic infiltration were tested. These factors were: immunological compatibility (auto- vs. allotransplant), donor age (2 vs. 9 dph), time the transplant spent in holding media (15 vs. 60 mins) and media temperature (RT vs. 4°C). As shown in Table 1, these factors were tested across seven groups while an unoperated group served as control (*n* = 5 poults/group). All poults were euthanized 6 days post-surgery (8 dph).

**Table 1:** Study 1 treatment groups.

**Treatment**	**Transplant type**	**Donor age (dph)**	**Time in HM (temperature)**
A	UO	–	–
A	Auto	2	15 min (RT)
B	Allo	2	15 min (RT)
C	Allo	2	15 min (4°C)
D	Allo	2	60 min (4°C)
E	Allo	9	15 min (RT)
F	Allo	9	15 min (4°C)
G	Allo	9	60 min (4°C)

*Autotransplant (auto) refers to an ovary which was removed, and placed back into the same recipient poult, whereas allotransplant (allo) refers to an ovary which was placed in a different poult. There were five recipient poults in each group (n = 5/group)*.

*UO, unoperated; dph, days post-hatch; RT, room temperature*.

#### Study 2

Based on Study 1 results, donor ovarian tissue was hereafter kept in holding media for 60 mins at 4°C. A total of nine immunosuppressive treatment protocols were tested as shown in Table 2. A tenth unoperated group that did not receive any immunosuppressant was used as control group. Factors that were tested included immunosuppressant type, dose, administration route, and administration schedule. For the first immunosuppressant MFM (Cellcept, Roche, Mississauga, ON, Canada), daily doses of 100 or 150 mg/kg/day were administered by crop gavage (**CG**) with treatment starting pre-surgery at 1 dph and continuing until 15 dph. The manufacturer recommendations state oral administration on an empty stomach. In previous studies, MFM was administered orally to birds that had been fed (11, 16, 17, 23). This is most likely because fasting young birds' post-surgery is not practical and discouraged, however, it cannot be ruled out that this may reduce the effectiveness of the drug. Consequently, we also administered MFM at 100 mg/kg/day *via* subcutaneous injection (**SC**) between the scapulas, beginning this daily treatment at 2 dph and continuing through to 15 dph. The second immunosuppressant CY (Procytox, Baxter, Mississauga, ON, Canada) was administered *via* intramuscular injection (IM) into the left pectoral muscle at 200 mg/kg. As a chemotherapeutic agent, CY has been shown to reduce the number of germ cells in mice ovaries (24, 25). Treatment with this drug while the transplant was developing post-surgery would therefore appear counterproductive, so CY was only given once pre-surgery at 1 dph. The third and final immunosuppressant, CsA (Sandimmune, Novartis, Mississauga, ON, Canada) was administered daily SC between the scapulas at 12.5, 25 or 50 mg/kg/day. The CsA was packaged in 1 ml vials at a concentration of 50 mg/ml, which was diluted to 25 mg/ml before being injected into the birds. The 50 mg treatment was started either pre- (50^*^) or post-surgery (50), whereas the 12.5 and 25 mg/kg/day treatments were both started post-surgery. This is because giving CsA pre-surgery resulted in anesthetic complications, which were prevented when the treatments started post-surgery. All birds were euthanized at 14 days post-surgery. Although five poults were allocated to each treatment group (n = 5/group), a purposeful reduction was implemented for the MFM at 100 mg/kg/day *via* CG group due to a lack of observed effectiveness and, the CsA 50^*^ mg/kg/day group due to severe adverse effects. This was implemented as part of the requirements of Animal Utilization Protocol to prevent unnecessary surgeries and minimize levels of pain and distress.

**Table 2:** Study 2 treatment groups.

**Treatment**	**Immuno-suppressant**	**Aministration route**	**Dosage (mg/kg/day)**	**Treatment started (dph)**	**Treatment finsihed (dph)**	**Number of poults**
1	UO	–	–	–	–	5
2	NS	–	–	–	–	5
3	MFM	CG	100	1	15	3
4	MFM	CG	150	1	15	5
5	MFM	SC	100	2	15	5
6	CY	IM	200	1	1	5
7	CsA	SC	12.5	2	15	5
8	CsA	SC	25	2	15	5
9	CsA	SC	50	2	15	5
10	CsA	SC	50^*^	1	15	3

*As surgery occurred at 2 dph, poults which started treatment at 1 dph had immunosuppressant in their system upon surgery, whereas poults which had their treatment begin at 2 dph were given their first dose following surgery. There were three to six recipient poults in each group (n = 3 to 5/group)*.

* *Distinguishes the 9th and 10th treatment groups*.

*UO, unoperated; US, unsuppressed; MFM, mycophenolate mofetil; CY, cyclophosphamide; CsA, cyclosporin A; CG, crop gavage; SC, subcutaneous injection; IM, intramuscular injection; dph, days post-hatch*.

#### Study 3

The final study included three groups of five birds that were euthanized 5 weeks post-surgery (*n* = 5/group). The first group was treated with 25 mg/kg/day of CsA administered SC in four separate injection sites to minimize tissue trauma. These were the left and right scapulas, and the left and right inner thigh. Sites were rotated starting the day of surgery through to the 34th day post-surgery. The second group underwent surgery but did not receive CsA, and the last group was not operated on or treated with an immunosuppressant.

### Ovarian Transplantation

The procedure for ovarian transplantation in neonatal turkey poults followed the protocol described by Hall et al. (12) with the following anesthetic modification. The anesthetic protocol was changed to IM administration of butorphanol (2 mg/kg), and xylazine (4 mg/kg) into the left pectoral muscle. Poults were returned to the intensive care unit for 10 mins to allow for full sedation. They were then placed onto a heating pad, after which the interscapular feathers were shaved to permit SC alfaxalone (30 mg/kg) administration. This induced and maintained the poults on a surgical plane of anesthesia for ~30 to 45 mins.

During surgery the entire recipient ovary was removed, and two pieces of ovarian tissue were transplanted into each recipient, but the abdominal air sac membrane was not used to cover the transplants (12). For the autotransplants in Study 1 the ovary was removed dissected into two pieces (2 × 2 mm^2^), and then placed back into the same recipient poult. All other transplants mentioned were allotransplant referring to an ovary that was placed into a different poult. Five sets of grafts (two pieces of ovarian tissue each) were weighed before transplantation, as 8 dph fresh transplants, to be compared with weights when the allotransplants were collected later in Study 2.

### Blood Collection and Leukocyte Count

For each recipient in Study 2 and 3 the total leukocyte concentration and the absolute concentration of circulating lymphocytes, heterophils, eosinophils, basophils, monocytes were calculated and compared between groups. Blood samples (0.3 ml) were collected from the jugular (Study 2) or wing vein (Study 3) just prior to euthanasia and were placed into a neonatal EDTA blood vial (Microvette CB 300 K2E, Starstedt AG and Co., Numbrecht, Germany). To determine the concentration of specific leukocytes a heterophil/eosinophil count was conducted using a hemocytometer, followed by a differential leukocyte count of 100 cells on a blood smear. For hemocytometer counts, 25 μl of blood was added to 775 μl of Eosinophil stain (ENG Scientific Inc., New Jersey, USA) resulting in a 32-fold dilution. The mixture was gently mixed on a shaker for 5 mins and then 10 μl of the mixture was loaded into each side of a Neubauer hemocytometer (Fisher Scientific, Hampton, NH, USA) and cells were allowed to settle for 2 mins. All purple-stained cells (heterophils and eosinophils) were counted within the 1 by 1 mm square on each side of the hemocytometer, and the average was used to estimate the heterophil and eosinophil concentration within the 0.1 mm^3^ sample. This value was multiplied by the dilution factor, 32, to give the combined concentration of heterophils and eosinophils.

To prepare the blood smear, 5 μl of blood was pipetted onto a microscope slide, a push preparation was made, and rapidly dried using a fan. The smear was stained using the Fisher HealthCare™ PROTOCOL™ Hema 3™ Fixative and Solutions. Based on the preference of the clinical pathologist, blood films received two dips in fixative and three dips in each of staining solutions I and II. Stained smears were air dried and coverslipped. Each blood smear was evaluated at low power for red cell density and white blood cell distribution. A differential white blood cell count was performed in areas free of cell lysis at 400 × or 1,000 × magnification until 100 leukocytes had been counted. Cells enumerated included: heterophils, lymphocytes, monocytes, basophils, and eosinophils. One of the birds in the CsA 25 mg/kg/day treatments in the second study had two additional counts performed and averaged due to inconsistent leukocyte proportions.

The total leukocyte count was determined by dividing the hemocytometer count by the total value of heterophils, and eosinophils expressed as a percent. The absolute count for each type of leukocyte was calculated using the total leukocyte concentration multiplied by the percent they made up within the leukocyte differential count. Each bird's absolute leukocyte concentration and the absolute concentration of circulating lymphocytes, heterophils, eosinophils, basophils, monocytes were compared between treatment groups.

### Organ Sampling and Relative Weights

In Study 1, ovaries were removed from recipient poults, fixed in Bouin's fixative for 1 h at RT, followed by an additional 23 h at 4°C (26). For birds in Studies 2 and 3, the ovary, thymus, spleen, and bursa of Fabricius were collected and weighed. Ovaries were then fixed as in Study 1. The ovary weight for birds in Study 2 was then compared to the 8 dph fresh transplant weight to determine if the tissue had increased in size during the study. In Study 3, ovary weights were compared between the immunosuppressed and unsuppressed birds. The lymphatic organ weights were divided by the body weight of the poult and expressed as relative weights.

### Ovary Processing, Staining, and Imaging

After the ovaries were fixed, tissues were washed with PBS, transferred to a tissue cassette, and stored in 70% ethanol at 4°C until processing. Samples were then dehydrated and cleared with xylene, embedded in paraffin blocks, and sectioned at a 5 μm thickness, parallel to the sagittal plane (13, 26) using a Finesse ME microtome (ThermoShandon, Cheshire, UK). Four serial sections were collected onto a slide and then 20 sections (100 μm) were discarded before collecting another four serial sections. This was repeated until the whole transplant was sectioned. In all studies, two random slides were selected from each ovary for CD3 and PAX5 immunohistochemical staining and analysis. In the third study, four slides were also randomly selected and stained with hematoxylin and eosin (**H&E**) to assess follicular size and density. The first section on each of the H&E slides were imaged using a Leica DM 5000B light microscope (Leica, Wetzlar, Germany) equipped with a B-Series LED light source (ScopeLED, Richmond, CA, USA) for sequential red/green/blue imaging. Images were captured using a Hamamatsu Orca-Flash 4 camera (Hamamatsu Photonics, Hamamatsu City, Japan) at 40 × magnification and processed using Volocity (ver. 6.3.1; Quorum Technologies, Guelph, ON, Canada) over the entire section; these images were subsequently stitched together to provide a large, high-resolution image of the first section. All chemicals were purchased through Fisher Scientific (Hampton, NH, USA), unless otherwise mentioned.

### Immunohistochemistry for CD3 and PAX5

Immunohistochemistry for cytoplasmic CD3 and nuclear PAX5 antigens were performed at the Animal Health Laboratory, University of Guelph (an American Association of Veterinary Laboratory Diagnosticians accredited laboratory), using an automated staining instrument (Dako Autostainer, Dako/Agilent, Ontario, Canada). The immunohistochemical reaction for CD3 followed the protocol detailed by Hall et al. (12). For PAX5, after manual deparaffinization and rehydration, the sections were treated with 3% hydrogen peroxide to quench endogenous peroxidase activity. Heat-induced epitope retrieval was carried out using an EDTA buffer (pH 9) and a pressure cooker device (PT Link, Dako). Primary antibodies against PAX5 (mouse monoclonal, clone 24, Biocare Medical) were diluted 1:50, and incubated at RT for 30 mins. Slides were then incubated with a dual anti-mouse / anti-rabbit anti-IgG horseradish peroxidase–linked polymer (EnVision FLEX, Dako) for 30 mins at RT, and reactions were visualized using Nova Red chromogen (Vector Laboratories, Burlington, ON, Canada). Slides were counterstained with hematoxylin. For CD3 and PAX5 immunohistochemical analysis, thymus, and bursa of Fabricius were used as positive controls, respectively. Negative controls were incubated in the absence of primary antibody. Sections were imaged using the same microscope and camera as described previously.

### Percent of CD3 and PAX5 Immunostained Area

The percent of positive immunostained area for T and B cell markers were evaluated to determine the extent of graft infiltration as detailed in Hall et al. (12). In the current study, PAX5 was used instead of MUM-1 as a nuclear B cell marker, as PAX5 is more generally expressed in avian B cells (27), whereas the former is expressed in late-stage B cells, such as plasma cells (28). In this study, three images were captured randomly throughout each transplant sample. This meant that for each sample the approximate area analyzed was 316,875 μm^2^. Since PAX5 is also a nuclear marker, the images were analyzed in a similar manner to MUM-1 (12). For CD3, the total percent of positive cytoplasmic area from all three images was divided by the total percent of cytoplasm to provide a percent of positive cytoplasmic area. For PAX5, the total percent of positive nuclear area from all three images was divided by the percent of total nuclear area, corresponding to the percent of positive nuclear area.

### Follicular Diameter, and Density

In Study 3, to determine if immunosuppression permitted transplants to mature and develop normally, the size and density of ovarian follicles were compared to unoperated birds. The calculation of follicular size and density followed the methods described by Hall et al. (26), with 30 follicles randomly selected and measured, and the density calculated using four slides (four serial sections per slide) per ovary. Both the size of the 30 follicles, and the density from the four slides were averaged, giving the specific follicle size and density for each ovary.

### Statistical Analysis

Statistical analyses were performed using SPSS 25.0 for Mac (SPSS Inc., Chicago, IL). All data are presented as mean ± standard error of the mean (SEM). The CD3 and PAX5 percent immunostained area data was not normally distributed, consequently a log transformation was performed before analysis. Normality and equal variance of all data was evaluated by residual plots and Levene's tests, respectively. A four-way ANOVA was used to analyze the CD3 and PAX5 immunostained data for Study 1. The four factors were type of transplant (auto- vs. allograft), donor age (2 vs. 9 dph), time in holding media (15 vs. 60 mins), and temperature (RT vs. 4°C). Significance of these factors and their interactions were reported. A one-way ANOVA followed by a *post-hoc* Tukey test was used for all other analyses throughout this study, except for ovary weight and follicular size in Study 3, this was analyzed using an independent t-test, as there were only two groups compared. Significance for all tests was set at (*P* ≤ 0.05).

## Results

### Surgery Survival Rate

The total number of surgeries performed during these studies was 113, with 108 (96%) recipients surviving the roughly 30 min procedure. Of the recipients who survived the surgery, 106 or 98% (106/108), regained consciousness within 1 to 2 h. Therefore, from the start of surgery to regaining of consciousness, the total survival rate was 94%. The only treatment group that reacted poorly to the surgery were the recipient poults that received CsA 50^*^ mg/kg/day. Out of the six surgeries performed with this immunosuppressant treatment, two poults died during surgery, and one died during recovery. It appeared that the birds experienced a negative drug interaction between CsA and one of the anesthetics or analgesics. Thereafter, CsA was given to birds only post-surgery for the other CsA treatments.

### Study 1

#### Lymphocytic Infiltration

**Figure 1:**
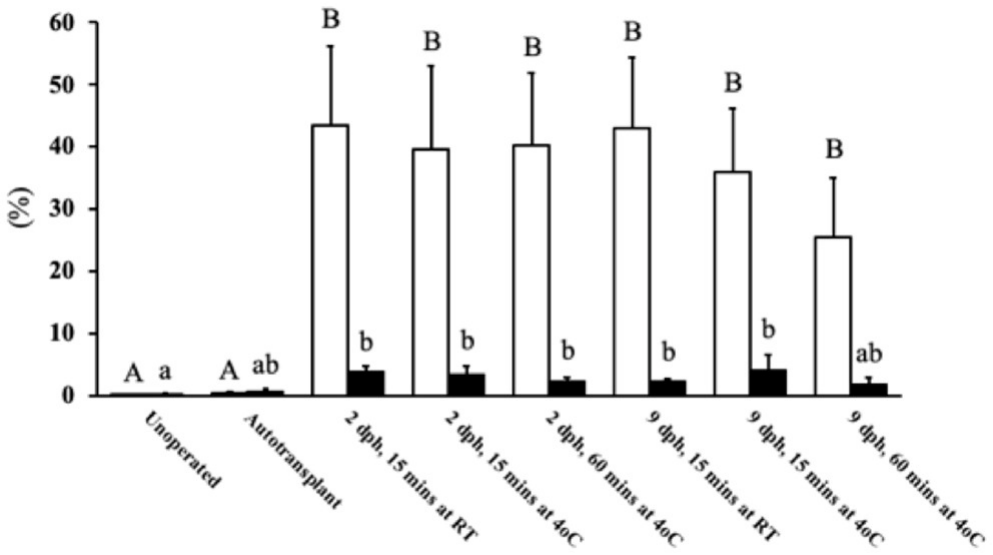
T and B cell levels within transplants from Study 1. After 6 days post-surgery, there were high levels of T cells within the transplants for all treatment groups compared to the unoperated and autotransplant groups; there was also an increase in B cells but not to the same degree. The open bars show the percent of CD3 immunostained cytoplasm within the transplants, used to estimate the amount of T cells; whereas the solid black bars show the percent of PAX5 immunostained nuclei used to estimate the number of B cells. Tissue was collected from unoperated control birds at 8 dph. For the autotransplants and allotransplants, the tissue was collected at 6 days post-surgery (8 dph). There was no difference in T and B cell levels within the allotransplants based on donor age (2 vs. 9 dph), the time the tissue spent in holding media (15 vs. 60 mins), or the temperature of the media (RT vs. 4°C). Data are means ± SEM of *n* = 5 . Statisitcs: one-way ANOVA followed by a *post-hoc, P* ≤ 0.05. ^A, B^means for percent of CD3 with no common superscript differ significantly. ^a, b^Means for percent of PAX5 with no common superscript differ significantly. dph, days posthatch; RT, room temperature.

There was no significant difference in percent of positive CD3 cytoplasmic area and percent of positive PAX5 nuclear area between the unoperated and autotransplant groups (Figure 1). However, ovaries from allotransplants showed a significantly stronger immunohistochemical staining for both CD3 (25–40% of the cytoplasmic area; *P* < 0.05) and PAX5 (2–4% of the nuclear area; *P* < 0.05) compared to the autotransplant group. Age of donor, time in the holding media, and temperature of the media, along with their interactions: age^*^time, and age^*^temperature had no significant effect on the percent of CD3 or PAX5 positive areas, respectively. Although not statically significant, the 9 dph transplants kept in holding media at 4°C for 60 mins displayed the lowest mean CD3 positive cytoplasmic area. Thus, to potentially maximize graft health and survival, this treatment was applied in Studies 2 and 3.

### Study 2

#### Poult Health

At the start of the second study all groups weighed the same, with poults weighing between 55 and 62 g (Table 3). At 14-days post-surgery there was no difference in poult body weight between the unoperated group and the unsuppressed, MFM, and CsA 12.5 mg/kg/day treatment groups. However, poults in the CY, CsA 25, CsA 50, and CsA 50^*^ mg/kg/day treatments had lower (*P* < 0.05) body weights compared to the unoperated group. With poults in the CsA 50^*^ mg/kg/day having the lowest average body weight. For the CY and CsA 25 mg/kg/day, based on the observation of activity level, this lower weight did not appear to affect overall poult health. However, based on anecdotal observations, the CsA 50^*^ mg/kg/day treated birds appeared to have a reduced activity level toward the end of the study. Birds in this group appeared lethargic and were excreting clear fluid from their vents, leading to soiling of the vent and abdomen. Samples of the fluid were not kept for analysis. Upon examination of the injection site during the post-mortem evaluation, large areas of scaring with large amounts of heterophilic inflammation were observed.

**Table 3:** Poult body weight (g) from 0 to 35 days post-surgery, from Study 2 and 3.

**Treatment (mg/kg/day)**	**Days post-surgery**					
	**0**	**7**	**14**	**21**	**28**	**35**
Unoperated	55 ± 1^a^	189 ± 4^c^	389 ± 07^e^	688 ± 12^b^	1,165 ± 26^b^	1,822 ± 40^c^
Unsuppressed	62 ± 1^a^	182 ± 5^c^	403 ± 17^e^	693 ± 30^b^	1,144 ± 46^b^	1,630 ± 51^b^
MFM (100) CG	55 ± 1^a^	166 ± 5^bc^	381 ± 10^de^	–	–	–
MFM (150) CG	56 ± 1^a^	171 ± 5^bc^	395 ± 14^de^	–	–	–
MFM (100) SC	60 ± 2^a^	181 ± 7^c^	397 ± 18^de^	–	–	–
CY 10 mg, IM	60 ± 2^a^	131 ± 9^a^	295 ± 16^ab^	–	–	–
CsA (12.5) SC	58 ± 1^a^	173 ± 5^bc^	357 ± 09^cde^	–	–	–
CsA (25) SC	58 ± 2^a^	169 ± 4^bc^	336 ± 03^bcd^	568 ± 12^a^	898 ± 18^a^	1,296 ± 30^a^
CsA (50) SC	60 ± 3^a^	160 ± 8^ac^	297 ± 16^abc^	–	–	–
CsA (50*) SC	56 ± 1^a^	144 ± 6^ab^	239 ± 04^a^	–	–	–

* *Indicates the treatment where 50 mg/kg/day of cyclosporin A was started pre-surgery. Weights are expressed as mean ± SEM or n = 3 to 5 per treatment. Statisitcs: one-way ANOVA followed by a post-hoc, P ≤ 0.05*.

a−e *Means with no common superscript in the same column differ significantly*.

*MFM, mycophenolate mofetil; CY, cyclophosphamide; CsA, cyclosporin A; CG, crop gavage; SC, subcutaneous injection; IM, intramuscular injection*.

#### Transplant Weight, and Lymphatic Organ Relative Weight

The relative weight of the bursa of Fabricius was smallest (*P* < 0.05) in the CY treatment, compared to all the other treatments (Table 4). For the thymic relative weight, only two treatments were significantly different from the others. The CY treatment had the lowest overall average thymic weight, which was significantly lower (*P* < 0.05) compared to the CsA 12.5, and CsA 25 mg/kg/day treatments. The other was the CsA 12.5 mg/kg/day treatment itself, which also had a higher (*P* < 0.05) value compared to the unoperated treatment. For splenic relative weights the CsA 25 mg/kg/day treatment had the lowest (*P* < 0.05) weight compared to the MFM 150, CsA 50, and CsA 50^*^ mg/kg/day, however, there was no significant difference compared to the unsuppressed group. Again, with no trend identified related to immunosuppressant, the biological relevance of these differences seems negligible.

**Table 4:** Bursal, thymic, and splenic relative weights (%), from Study 2 and 3.

**Treatment (mg/kg/day)**	**14 days post-surgery**			**35 days post-surgery**		
	**Bursa (RW)**	**Thymus (RW)**	**Spleen (RW)**	**Bursa (RW)**	**Thymus (RW)**	**Spleen (RW)**
Unoperated	0.137 ± 0.009 ^bc^	0.255 ± 0.026^ab^	0.087 ± 0.007^ab^	0.105 ± 0.005^a^	0.257 ± 0.018^a^	0.098 ± 0.004^a^
Unsuppressed	0.137 ± 0.008 ^bc^	0.310 ± 0.009^ac^	0.091 ± 0.004^ab^	0.109 ± 0.004^a^	0.260 ± 0.013^a^	0.103 ± 0.005^a^
MFM (100) CG	0.119 ± 0.014^b^	0.308 ± 0.005^ac^	0.098 ± 0.004^ab^	–	–	–
MFM (150) CG	0.120 ± 0.011^bc^	0.317 ± 0.023^ac^	0.114 ± 0.009^b^	–	–	–
MFM (100) SC	0.098 ± 0.014^b^	0.322 ± 0.019^ac^	0.091 ± 0.004^ab^	–	–	–
CY 10 mg, IM	0.046 ± 0.004^a^	0.250 ± 0.018^a^	0.085 ± 0.010^ab^	–	–	–
CsA (12.5) SC	0.167 ± 0.008^c^	0.352 ± 0.020^c^	0.079 ± 0.009^ab^	–	–	–
CsA (25) SC	0.137 ± 0.008^bc^	0.342 ± 0.016^bc^	0.061 ± 0.004^a^	0.124 ± 0.006^a^	0.256 ± 0.018^a^	0.091 ± 0.007^a^
CsA (50) SC	0.104 ± 0.012^b^	0.284 ± 0.015^ac^	0.116 ± 0.015^b^	–	–	–
CsA (50^*^) SC	0.096 ± 0.010^b^	0.323 ± 0.015^ac^	0.108 ± 0.010^b^	–	–	–

* *Indicates the treatment where 50 mg/kg/day of cyclosporin A was started pre-surgery. Relative weights (RW) are expressed as mean ± SEM of n = 3 to 5 per treatment. RW was defined as organ weight (mg) / body weight (mg) ×100. Statisitcs: one-way ANOVA followed by a post-hoc, P ≤ 0.05*.

a−c *Means with no common superscript in the same column differ significantly*.

*MFM, mycophenolate mofetil; CY, cyclophosphamide; CsA, cyclosporin A; CG, crop gavage; SC, subcutaneous injection; IM, intramuscular injection*.

**Figure 2:**
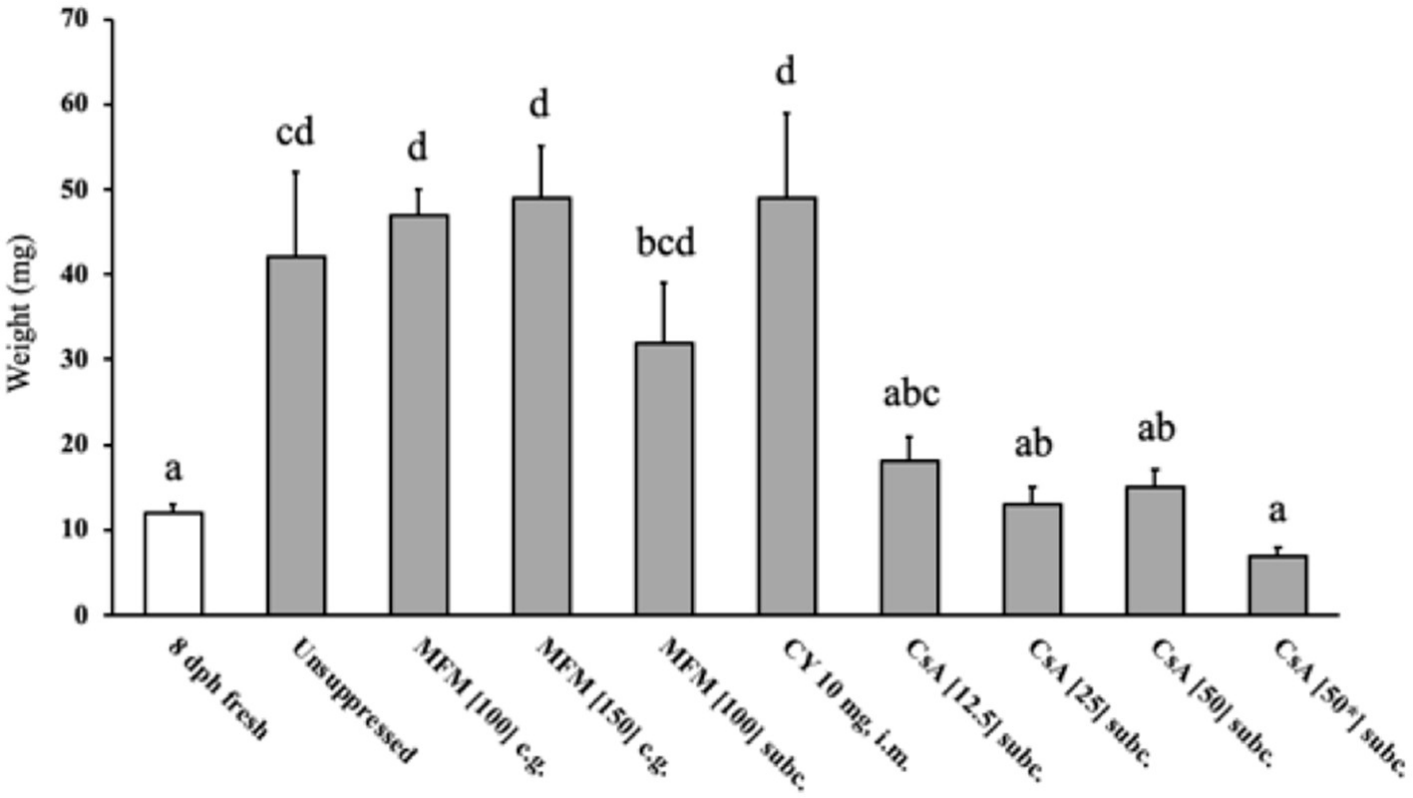
Weight of ovarian transplants 14 days post-surgery. The 8 dph fresh tissue (open bar) represents the size of the two pieces of ovarian grafts when transplantation occurred, all other weights (grey bars) indicate the transplant size after the 14 days post-surgery. The transplants within birds treated with CsA did not increase in weight during the 14 days, whereas the unsuppressed, MFM and CY treated birds saw their ovaries significantly increase in weight. * Indicates the treatment where 50 mg/kg/day of cyclosporin A was started pre-surgery. Data are means ± SEM of *n* = 3 to 5. Statistics: one-way ANOVA followed by a *post-hoc, P* ≤ 0.05. ^a−*d*^Means with no common superscript differ significantly. dph, days posthatch; MFM, mycophenolate mofetil; CY, cyclophosphamide; CsA, cyclosporin A; CG, crop gavage; SC, subcutaneous injection; IM, intramuscular injection.

To assess the growth of the transplants at 14 days post-surgery the weights were compared to the 8 dph pre-surgery transplant weight (Figure 2), and not the unoperated group as these poults possessed a whole ovary which would prevent a fair comparison. The unsuppressed, MFM, and CY treatment groups showed transplant growth (*P* < 0.05) during the 14 days, which equated to roughly a three-to-five-fold increase in size, whereas the CsA treatment groups did not display any growth.

#### Circulating Lymphocytes and Total Leukocytes

No difference in the number of circulating eosinophils, basophils, and monocytes was observed between treatment groups (Table 5). Although absolute heterophils differed between experimental groups, the differences did not appear to be associated with an immunosuppressant or route of administration, however, the lowest number was seen in the CsA 50^*^ mg/kg/day group, and the highest number was seen in the MFM 100 mg/kg/day SC treatment. Conversely, for lymphocytes, treatment had a significant effect, as the CsA groups displayed lower means than all the other treatment groups in a dose dependent manner with the two CsA 50 mg/kg/day treatment groups reaching statistical significance (*P* < 0.05) compared to the unsuppressed, and MFM 100 mg/kg/day CG treatments. This reduction in lymphocytes was also seen in the total leukocyte count, which was lower in the two CsA 50 mg/kg/day treatments. Interestingly, although not statistically significant, the highest number of lymphocytes was observed in the unsuppressed, MFM 100 mg/kg/day CG and CY treatments.

**Table 5:** Concentration of circulating leukocytes and their subsets, from Study 2.

**Treatment (mg/kg/day)**	**Lymphocyte**	**Heterophil**	**Eosinophil**	**Basophil**	**Monocyte**	**Leukocyte**
Unoperated	7,996 ± 721^ac^	8,486 ± 276^abcd^	506 ± 162^a^	914 ± 336^a^	2,252 ± 311^a^	20,154 ± 1,239^abc^
Unsuppressed	10,549 ± 2,531^bc^	7,055 ± 1,462^abc^	572 ± 159^a^	1,544 ± 376^a^	1,524 ± 267^a^	21,244 ± 2,729^bc^
MFM (100) CG	16,940 ± 7,799^c^	9,418 ± 1,451^abcd^	1,088 ± 335^a^	1,616 ± 944^a^	2,315 ± 1,050^a^	31,378 ± 7,835^c^
MFM (150) CG	8,529 ± 1,026^ac^	7,952 ± 1,049^abcd^	592 ± 180^a^	1,506 ± 607^a^	1,335 ± 323^a^	19,915 ± 2,506^abc^
MFM (100) SC	7,326 ± 1,187^ac^	15,460 ± 1,749^d^	1,020 ± 221^a^	864 ± 191^a^	2,491 ± 265^a^	27,161 ± 2,438^bc^
CY 10 mg, IM	10,357 ± 2,821^ac^	6,422 ± 597^ab^	1,226 ± 318^a^	184 ± 48^a^	2,614 ± 428^a^	20,787 ± 3,069^abc^
CsA (12.5) SC	5,968 ± 1,372^ab^	12,238 ± 1,543^bcd^	434 ± 147^a^	1,238 ± 270^a^	3,576 ± 690^a^	23,455 ± 2,149^abc^
CsA (25) SC	2,780 ± 822^ab^	12,726 ± 1,119^cd^	298 ± 155^a^	719 ± 184^a^	1,270 ± 182^a^	17,793 ± 1,708^abc^
CsA (50) SC	1,994 ± 492^a^	6,995 ± 2,018^abc^	301 ± 148^a^	702 ± 390^a^	3,081 ± 743^a^	13,073 ± 2,663^ab^
CsA (50^*^) SC	1,863 ± 501^a^	4,514 ± 956^a^	179 ± 93^a^	761 ± 291^a^	3,185 ± 428^a^	10,502 ± 1,770^a^

* *Indicates the treatment where 50 mg/kg/day of cyclosporin A was started pre-surgery. Cell concentrations are expressed as mean ± SEM of n = 3 to 5 per treatment. Statisitcs: one-way ANOVA followed by a post-hoc, P ≤ 0.05*.

a−d *Means with no common superscript in the same column differ significantly*.

*MFM, mycophenolate mofetil; CY, cyclophosphamide; CsA, cyclosporin A; CG, crop gavage; SC, subcutaneous injection; IM, intramuscular injection*.

#### Lymphocytic Infiltration

**Figure 3:**
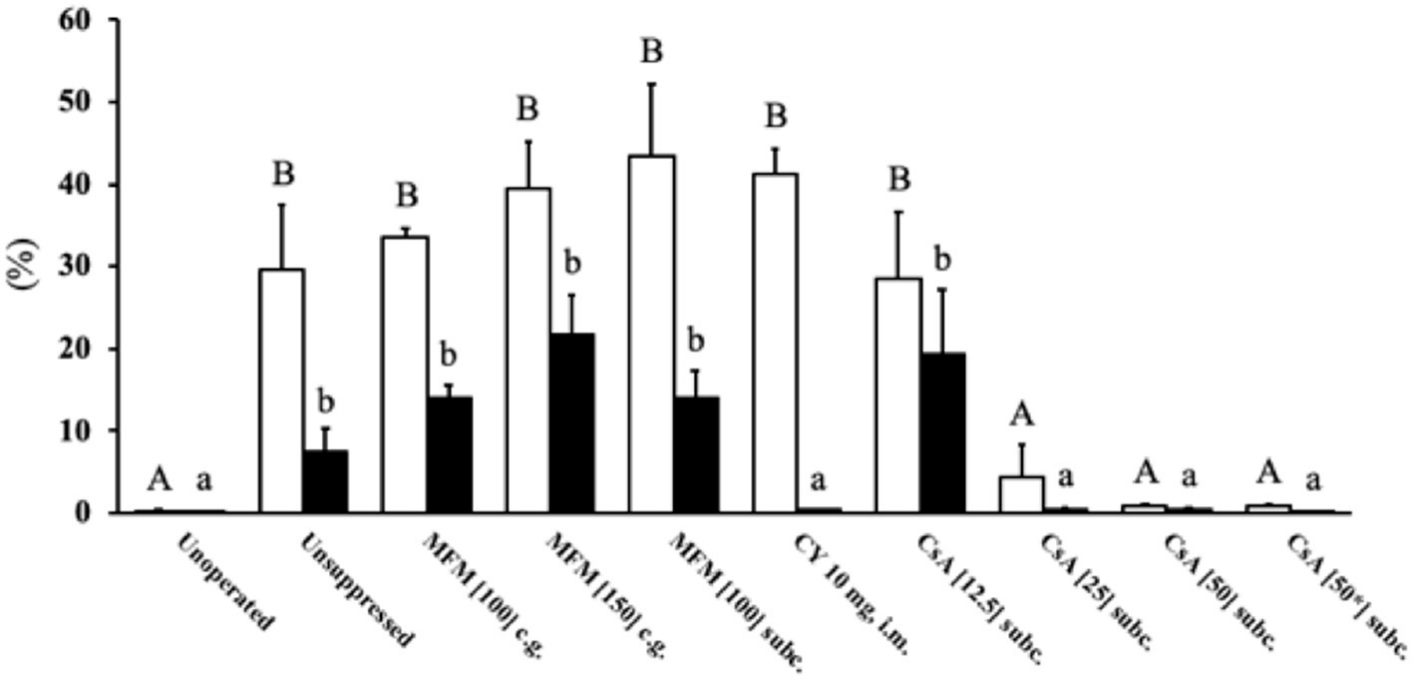
T and B cell levels within transplants from Study 2. After 14 days post-surgery, there were high levels of T cells within the unsuppressed, MFM, CY and CsA 12.5 mg/kg/day treatment groups, compared to the unoperated and CsA 25 and 50 mg/kg/day groups. The only difference between the T and B cell levels was that in the CY treatment were low levels of B cells were observed. The open bars show the percent of CD3 immunostained cytoplasm which was used to estimate the amount of T cells, whereas the solid black bars show the percent of PAX5 immunostained nuclei used to estimate the number of B cells. The unoperated tissue was collect from birds at 16 dph. For all other treatments, the tissue was collected at 14 days post-surgery (16 dph). The dose (mg) of the drug indicates the amount given per kg per day. * Indicates the treatment where 50 mg/kg/day of cyclosporin A was started the pre-surgery. Data are means ± SEM of *n* = 3–5. Statistics: one-way ANOVA followed by a *post-hoc, P* ≤ 0.05. ^A, B^Means for percent of CD3 with no common superscript differ significantly. ^a, b^Means for percent of PAX5 with no common superscript differ significantly. dph, days posthatch; MFM, mycophenolate mofetil; CY, cyclophosphamide; CsA, cyclosporin A; CG, crop gavage; SC, subcutaneous injection; IM, intramuscular injection.

There was a higher (*P* < 0.05) percent of CD3 positive cytoplasmic area in the unsuppressed, MFM, CY, and CsA 12.5 mg/kg/day treatments compared to the unoperated birds (Figure 3), with these high values ranging between 30 and 40%. Conversely, no significant difference in percent of CD3 positive cytoplasm was observed between unoperated birds and the CsA 25, 50, or 50^*^ mg/kg/day treatments, with these percent positive cytoplasmic area values ranging between 0.1 and 4%. This pattern was similar for the percent of PAX5 positive nuclear area, with higher levels (*P* < 0.05) in the unsuppressed, MFM, and CsA 12.5 mg/kg/day treatments, compared to the unoperated birds, with values ranging between 10 and 20% of the total positive PAX5 area. However, there was no significant difference between the unoperated and CY treatment. Just as for CD3, there was no significant difference in the percentage of positive PAX5 nuclear area between the unoperated, CsA 25, 50 and 50^*^ mg/kg/day treatments. For these groups the positive PAX5 nuclear area values ranged between 0.01 and 0.2%. Based on these results, CsA 25 mg/kg/day treatment appeared to be the best immunosuppressant and was selected for Study 3.

### Study 3

#### Body Weight and Health

At 5 weeks post-surgery the poults in the unoperated group had the highest (*P* < 0.05) body weight, compared to the unsuppressed group (Table 3). The immunosuppressed group had a lower (*P* < 0.05) body weight, compared to the unsuppressed group, at 5 weeks post-surgery. The problems which were seen with the CsA 50 mg/kg/day groups in Study 2, e.g., skin damage and fluid discharge from the vent, were also present by the 5th week with the CsA 25 mg/kg/day treatment group: however, the skin damage was not as severe, and the birds did not appear to have a reduced activity level as observed in Study 2.

#### Circulating Lymphocytes

After 35 days of CsA 25 mg/kg/day treatment, the recipients had fewer (*P* < 0.05) circulating lymphocytes than either the unsuppressed group, or the unoperated group (Table 6). This decrease was reflected in the total leukocyte count which was also lower (*P* < 0.05) in the suppressed group compared to the unsuppressed, or the unoperated group. There was no difference in the circulating numbers of heterophils, eosinophils, basophils, or monocytes among treatment groups.

**Table 6:** Concentration of circulating leukocytes and their subsets, from Study 3.

**Treatment (mg/kg/day)**	**Lymphocyte (/ul)**	**Heterophil (/ul)**	**Eosinophil (/ul)**	**Basophil (/ul)**	**Monocyte (/ul)**	**Leukocyte (/ul)**
Unoperated	16,464 ± 1,499^b^	7,881 ± 797^a^	899 ± 297^a^	1,642 ± 475^a^	2,145 ± 540^a^	29,031 ± 1,903^b^
Unsuppressed	12,385 ± 1,805^b^	8,091 ± 397^a^	613 ± 184^a^	1,339 ± 264^a^	1,922 ± 577^a^	24,349 ± 2,401^b^
CsA (25) SC	2,786 ± 481^a^	9,084 ± 607^a^	276 ± 47^a^	971 ± 189^a^	2,087 ± 358^a^	15,204 ± 860^a^

*Cell concentrations are expressed as mean ± SEM with n = 5. Statisitcs: one-way ANOVA followed by a post-hoc, P ≤ 0.05*.

a, b *Means with no common superscript in the same column differ significantly*.

*CsA, cyclosporin A; SC, subcutaneous injection*.

#### Lymphocytic Infiltration, and Ovary Follicle Density

**Figure 4:**
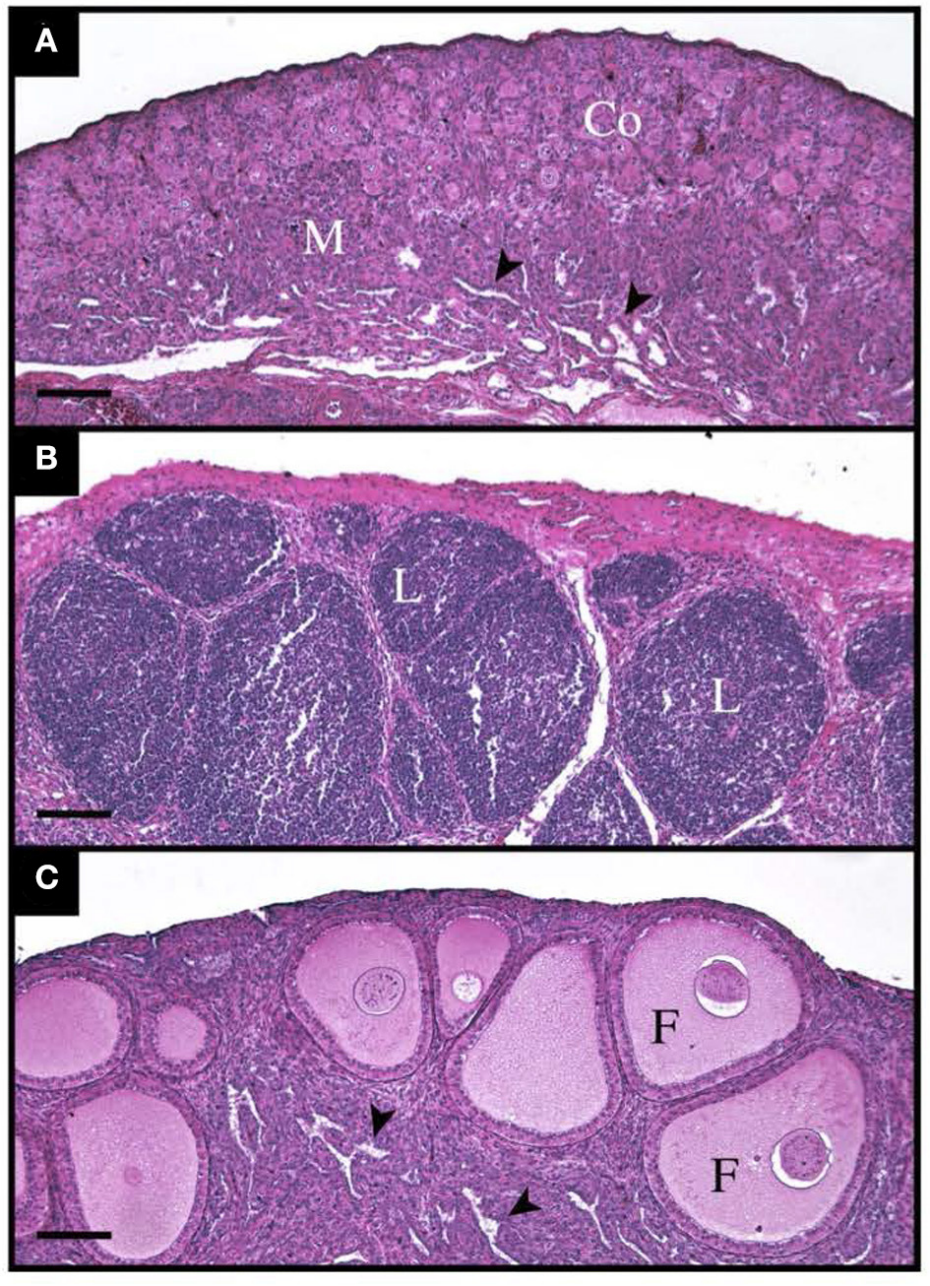
Histological appearance of tissue before, and 35 days post transplantation with and without immunosuppressants. **(A)** Shows 8-day old ovarian tissue before transplantation with a clear cortex (Co) and medulla (M) with lacunar channels (arrowheads) visible. **(B)** Shows an ovarian transplant 35 days post-surgery from an unsuppressed recipient. The entire graft is infiltrated by lymphocytic masses (L). **(C)** Also shows a transplant 35-days post-surgery, however this poult was treated with Cyclosporin A at 25 mg/kg/day. Normal follicles (F) can be seen throughout the cortex and lacunar channels (arrowheads) within the medulla.

There was a stark difference in the overall appearance of transplants from suppressed recipients vs. unsuppressed (Figure 4). The transplants from unsuppressed recipients were larger (*P* < 0.05), had a higher percentage of CD3 immunostained cytoplasmic area and PAX5 immunostained nuclear area, and did not contain any follicles (Table 7). With these transplants resembling lymphatic tissue, more than ovarian tissue (Figure 4B). The suppressed group had a statistically similar CD3 positive cytoplasmic area, and PAX5 immunostained nuclear area, compared to the unoperated group (Table 7). The suppressed group also had similar follicular size, although there were fewer (*P* < 0.05) follicles present, compared to the unoperated group. It was clear that CsA 25 mg/kg/day was not only able to stop rejection (Figure 4C), but it was also able to allow the transplants to mature, when compared to the 8 dph fresh transplants, before transplantation (Figure 4A).

**Table 7:** Ovary weight and lymphocytic levels, along with follicle size and density from Study 3.

**Treatment (mg/kg/day)**	**Ovary weight (mg)**	**Percent CD3 immunostained cytoplasm (%)**	**Percent PAX5 immunostained nuclei (%)**	**Follicular size (um)**	**Follicular density (#/mm^**3**^)**
Unoperated	–	0.13 ± 0.03^a^	0.05 ± 0.01^a^	110 ± 19^a^	2,103 ± 709^c^
Unsuppressed	62 ± 11^b^	48.37 ± 7.78^b^	39.68 ± 11.68^b^	–	0 ± 0^a^
CsA (25) SC	33 ± 4^a^	9.62 ± 5.61^a^	4.93 ± 4.52^a^	130 ± 28^a^	936 ± 457^b^

*All values here are expressed as mean ± SEM with n = 5. Statisitcs: t-test for ovary weight and follicular size; one-way ANOVA followed by a post-hoc for immunostained cytoplasm and nuclei along with follicular density, P ≤ 0.05*.

a, b *Means with no common superscript in the same column differ significantly*.

*CsA, cyclosporin A; SC, subcutaneous injection*.

## Discussion

Understanding why poultry ovarian transplants have varying success rates is vital if the procedure is to be routinely used to revive biobanked tissue. In the domestic turkey, transplant age (13), and amount of recipient tissue left behind (12) were eliminated as possible causes for the low success rates. However, in the latter paper it was demonstrated that transplants became highly infiltrated with lymphocytes by 6 days post-transplantation, implying rejection by the host's immune system. Therefore, the goal of this project was to ascertain if lymphocytic infiltration could be reduced and if that reduction would permit normal transplant development. Condition of the tissue with respect to immunological compatibility and donor age, along with treatment of the tissue, time in, and temperature of holding media pre-surgery were all investigated. Additionally, the effectiveness of three immunosuppressive drugs at varying doses and routes of administration were evaluated.

With a novel anesthetic protocol and increased surgical skill, the surgical survival rate of 94% was 14% higher than we previously reported (12). The drug CsA was the only one to have a negative effect on the surgical procedure when administered the day before surgery and immediately prior to surgery. We propose two possible explanations, either the dose was too high before surgery, which weakened the poults, or the CsA had a negative interaction with either butorphanol, xylazine and alfaxalone, or a combination of these drugs. Thus, it would not be recommended to treat poults with CsA before surgery and instead only start treatment post-surgery.

In the first study, we determined whether lymphocytic infiltration could be reduced by improving immunological compatibility, increasing the age of the donor, or keeping the tissue in chilled holding media for an extended period, pre-surgery. Improving immunological compatibility was accomplished by performing autotransplantations and comparing their lymphocytic infiltration to that of allotransplants. Autotransplants of dispersed testicular cells in chickens had a high level of success (10), but the use of ovarian autotransplants have not been assessed. In this work the autotransplants showed significant reduction in T cell infiltration compared to the allotransplants, with the autotransplants having similar infiltration compared to the unoperated birds. There was, however, no difference in the B cell population between the auto and allotransplants, noting that this was the minor lymphoid cell type present. Since all transplants undergo varying amounts of ischemia and reperfusion damage, which activates an inflammatory immune response (29), it is possible that even an autotransplant might display some lymphocytic infiltration. In this study we can rule out this as a possible cause of our infiltration in turkey ovarian transplants, since the autotransplants had relatively no infiltration. Within the allotransplants, donor age, time in holding media, and temperature of the media had no effect on the level of lymphocytic infiltration. It is clear that immunological compatibility had the only, and a large effect, on reducing lymphocytic infiltration by 6 days post-transplantation. Since lymphocytic infiltration triggered by immunological incompatibility was observed by 6 days post-transplantation, this rejection could be classified as hyperacute or acute based on hyperacute and acute occurring immediately or within 2 days, respectively (30, 31). Because transplanted ovarian tissue was not examined histologically before day 6 post-transplantation, we cannot know what type of rejection predominated. However, as poults exhibited no hyperacute decompensation and didn't have any known prior exposure to the donor's cells, we assumed the rejection was acute in nature. This is the first time that acute rejection of transplanted gonadal tissue has been identified in poultry species, and the first time this type of immunological response has been detected after gonads were transplanted within the same breed. In fact, when gonads were transplanted between the same chicken line, a high level of success was achieved (14), while this was not the case here and in a previous turkey study (32).

In the second study, immunosuppressants showed varying effects on ovary and lymphoid organ size, peripheral blood lymphocyte numbers, and transplant infiltration. In poultry ovarian transplantations, MFM is the most used immunosuppressant (11, 16, 17, 23). In mammals, MFM works by reducing the amount of guanosine and deoxyguanosine nucleotides in T and B cells, which impedes proliferation in both lineages (33). In this study, at the standard dose (100 mg/kg/day) and delivery method (CG) MFM did not eliminate lymphocyte tissue infiltration or reduce their circulating concentration. In addition, increasing the dose, and administering it subcutaneously had no effect on these variables either. Therefore, MFM does not improve ovarian transplantation success rates in the domestic turkey. These findings corroborate findings in chickens, which found that MFM did not improve transplant survival over unsuppressed birds (14, 16) but differed to those observed in quail (17). Therefore, our findings support the possibility that MFM effectiveness for poultry ovarian transplantations is species-specific.

The next two drugs discussed have not been used in conjunction with poultry ovarian transplantation but have been shown to effectively suppress the avian immune system (19–22). The first drug, CY, works by disrupting DNA synthesis which disproportionately affects rapidly dividing cells, causing cell death. In young birds this drug causes degeneration of the bursa of Fabricius and lymphoid organs, which depend on bursal functionality (34), effectively inducing a chemical bursectomy (35). This preferential effect on B cells by CY specifically affects production of antibodies in response to foreign antigens (22). In the current study, CY-treated poults had a noticeable decrease in body weight by day 14 post-surgery, compared to the unsuppressed birds. This decrease was expected as it has been previously observed in turkeys and chickens (20, 22). When CY was administered to broiler chicks at a similar dose, bursal weight was reduced by 70% (20). This result was similar to our observation, although we did not see a reduction in circulating lymphocytes as previously reported in turkeys (36). However, in the latter study, circulating lymphocytes were monitored for 72 h after CY injection while, in our study, analyses occurred 15 days after the drug was administered, indicating that circulating lymphocyte numbers can recover. At the level of the transplant, CY had no effect on T cell infiltration, but did reduce B cell infiltration, however, this did not prevent abnormal growth of the transplants. Therefore, CY as a monotherapy is ineffective at preventing graft rejection and is thus not recommend.

The third drug assessed, CsA, works by selectively blocking the transcription of the interleukin-2 gene within T cells, thereby blocking cellular proliferation (37). This drug therefore preferentially effects T cells. In this study CsA treated poults had a decreased body weight compared to the unsuppressed birds. This result was expected based on previous studies in turkeys and chickens (20, 22). However, based on another study (18), the negative health effects we observed were not anticipated at the 50 mg/kg/day dose. Based on these deleterious effects, this dose would not be recommend for future studies. However, CsA at this high dose did reduce the number of circulating lymphocytes, which is, to the best of our knowledge, the first reported observation in any poultry species. All doses of CsA elicited a reduction in both T and B cell infiltration within the transplants. This finding matches a previous study (18), which demonstrated that CsA at a dose of 25 mg/kg/day was effective at permitting chicken skin graft survival. Coupled with our results, it reaffirms that at this dose, CsA can prevent acute rejection in both chickens and turkeys, by 14 days post-surgery.

For the final study, based on the low concentrations of lymphocytic infiltration and normal ovarian follicular development, CsA 25 mg/kg/day again permitted healthy transplant development, up to 35 days post-surgery. However, the density of ovarian follicles was decreased, which was expected as transplantation, on its own, decreases the number of follicles within the turkey ovary (13). Unfortunately, the deleterious effects noted in the CsA 50 mg/kg/day treatments in Study 2 were also seen here with a lower dose by 35 days post-surgery. Based on this observation, an alternative method of administration such as an osmotic pump should be investigated to minimize tissue damage. Unfortunately, we did not analyse the fluid discharge, observed under CsA treatment but, since one of the most common side effects of CsA treatment is nephrotoxicity (38), we believe this could have caused excessive fluid loss at the level of the kidneys due to kidney damage.

One morphological feature which wasn't evaluated but should be discussed is vascularization. Adequate vascularization is important to maintain ovarian function during all stages of development. It is assumed that the transplants were vascularized since transplants which do not have proper blood supply become necrotic within days (39). It is unclear if this blood supply would be adequate to maintain these ovaries to full maturation, however, from previous studies it is clear, that the blood supply is sufficient to allow chicken, quail, and duck ovaries to become reproductively active (16, 17, 23).

Across all three studies, one noticeable trend emerged: this being the increase in B cell infiltration over time within the transplants. Within all groups in Study 1, which were not treated with any immunosuppressant, B cell infiltration was between 2 and 4% by day 6 post-surgery. In the second study which took the unsuppressed birds to 14 days post-surgery, the percentage increased to 7%, and in the final study which took unsuppressed birds to 35 days post-surgery, that percentage increased to 40%. Conversely, T cell infiltration remained relatively high (around 40%) for all age groups. To the authors' knowledge, this is the first time a slow increase in B cell infiltration over time has been observed in transplanted organs in a poultry species.

Despite multiple attempts, success rates in ovarian transplantations in poultry species remain low and variable (11, 14, 16, 23). Our results suggest that acute rejection based on immunological incompatibility is the most likely cause. Thus, genetically matching donors and recipients based on their MHC II complexes might reduce the dose of immunosuppressants required. As we identified the cause of ovarian transplant failure in turkeys, along with a potential solution, future studies should focus on determining whether ovarian transplants can survive and develop to sexual maturity and whether donor-derived progeny can be produced.

For ovarian transplantation in the domestic turkey, lymphocytic infiltration (acute rejection) is clearly the major cause of transplant failure by 35 days post-transplantation. Here, it was demonstrated that immunological compatibility or an immunosuppressant were both able to overcome the acute rejection. The most widely used immunosuppressant MFM, with respect to ovarian transplantation between poultry breeds, was ineffective in our study. CY was able to reduce B cell infiltration, but not T cell infiltration, suggesting that it should not be used on its own. CsA was able to reduce lymphocytic infiltration and allowed for normal transplant maturation. However, by 35 days post-transplantation the CsA 25 mg/kg/day treated birds showed significant side effects negatively impacting health. Moving forward, an alternative administration route, with or without reducing the dose of CsA if donors and recipients are genetically matched, could eliminate these negative side effects. If immunological incompatibility is eliminated, CsA will still likely play an important role in allowing turkey ovarian transplants to develop normally after transplantation.

## Data Availability Statement

The raw data supporting the conclusions of this article will be made available by the authors, without undue reservation.

## Ethics Statement

The animal study was reviewed and approved by University of Guelph Animal Care Services—Canadian Council on Animal Care.

## Author Contributions

GH designed the experiment, performed the surgical procedure, analyzed the data, and wrote the original manuscript. JB-M performed the WBC and analyzed that portion of the data. JL and GB provided funding and helped with experimental design. BW procurred animals and advised on the surgical procedure. All authors edited early drafts and read and approved the final manuscript.

## Funding

Our group had two support agreements from the United States Department of Agriculture (support agreement: 58-8042-7-068-F and 58-8042-9-070-F), which was facilitated by JL, one of the coauthors. These agreements supported the PhD students salary along with operating expenses. GB was already awarded an NSERC Discovery Grant (RGPIN-2015-04766), with this award given to researchers to be able to support projects outside of their fields. This award also supported the PhD students salary.

## Conflict of Interest

The authors declare that the research was conducted in the absence of any commercial or financial relationships that could be construed as a potential conflict of interest.

## Publisher's Note

All claims expressed in this article are solely those of the authors and do not necessarily represent those of their affiliated organizations, or those of the publisher, the editors and the reviewers. Any product that may be evaluated in this article, or claim that may be made by its manufacturer, is not guaranteed or endorsed by the publisher.
